# Visual-motor involvement in autism spectrum disorder: could the stereopsis deficit affect motor coordination?

**DOI:** 10.3389/fpsyt.2023.1130185

**Published:** 2023-11-16

**Authors:** Rosa Longo, Francesca Allegrini, Elena Gusson, Roberta Morbio, Gianfranco Di Gennaro, Luigi Alberto Gozzi, Giorgio Marchini, Leonardo Zoccante

**Affiliations:** ^1^Ophthalmic Unit, Department of Neurosciences, Biomedicine and Movement Sciences, Integrated University Hospital of Verona, Verona, Italy; ^2^Child and Adolescent Neuropsychiatry Unit, Maternal-Child Integrated Care Department, Integrated University Hospital of Verona, Verona, Italy

**Keywords:** autism, stereopsis, visual acuity, visual motor perception, enhanced perceptual functions

## Abstract

**Purpose:**

Given the known difficulty in sensory processing and in motor skills in patients with Autism Spectrum Disorder (ASD), and since visual impairment could interfere with children’s behaviour, early detection and management of visual-motor difficulties are crucial. This exploratory study aims to evaluate the visual-motor status in a cohort of children affected by ASD.

**Methods:**

The records of patients affected by ASD and admitted between 2018 and 2022 to the Pediatric University Hospital of Verona were reviewed. Best Corrected Visual Acuity, cycloplegic refraction, stereopsis, convergence, complete ocular motility, strabismus examination, slit-lamp anterior segment examination and fundus ophthalmoscopy were collected and reviewed.

**Results:**

A total of 253 patients (203 ASD and 50 healthy controls) were included in the study. A higher number of total orthoptic defects were detected in the ASD group, in comparison with the control group. Specifically, a higher percentage of stereopsis deficit and convergence insufficiency was observed.

**Conclusion:**

In our cohort of children with ASD stereopsis deficit, convergence insufficiency and refractive errors are the most observed ocular conditions. These findings are consistent with the known alterations of motor skills and sensory processing in ASD. Moreover, our study supports the hypothesis that visual acuity is not compromised in children with ASD. As a result, a complete ophthalmic evaluation is highly recommended in children with ASD, to guarantee early detection and treatment of possible visual-motor defects.

## Introduction

Autism spectrum disorders (ASD) are a group of heterogeneous neurodevelopmental disabilities that share some features like atypical social interaction and behavioural impairment (1) and are described as a spectrum that could range from very mild to severe forms, with a variable impact on the quality of life (2). To date, several studies have been conducted on ASD, nevertheless, many factors such as aetiology, diagnosis and prevention remain uncertain. Central nervous system dysfunction is a probable causative hypothesis, manifesting with systemic involvement, including vestibular, auditory and visual systems (3, 4).

Patients with ASD could show learning, social and communication difficulties, repetitive behaviours and limited interests. These symptoms could interfere with children’s daily life, having an important impact on school, social and family activities (1, 5). Typical symptoms appear when children begin social interaction, usually before the age of 3 years (1, 2). Subtle signs of ASD could otherwise be present before the first year of life. One of these signs is anomalous eye contact, which usually replaces the physical interaction by the ninth month of age (6). The inability to have eye contact with people and the aversion to looking at faces are important suspect signs (7, 8). Often parents note that their children seem to avoid looking at them, or “look right through them.” In this scenario, the term implies the focus of the child within himself, and gaze aversion gives a limitation of interaction with other than self (7).

Since the oculomotor system is essential for the development of the voluntary behavior, and its maturation is considered as paradigmatic of brain maturational processes (9–11), these features raised the suspicion that ASD also included a visual disturbance, so different studies considered the visual deficits in ASD patients (3, 12–15). While visual acuity and binocular vision do not appear to be particularly affected in patients with ASD, there is an emerging pattern of subtle visuo-motor deficits in stereoacuity, accommodation and near point of convergence (16). Moreover, several studies have explored the impairment of sensory processing in ADS patients (4, 8). Many elements seem to be involved in this deficit: visual attention, visual-motor integration and altered visual perception, i.e., the preference for static stimuli, rich in detail, rather than for dynamic or social stimuli (4, 8, 17). All these elements are commonly referred to as visual behavior and they seem to be the real focus of visual difficulties in patients with ASD (10).

Given the known difficulty in sensory processing in patients with ASD, and since visual-motor impairment could interfere with children’s behaviour, stress levels and receptiveness to new information, the ophthalmologic evaluation plays a key role (18). Early detection and management of vision difficulties ensure early intervention, referring the child to the appropriate professionals (19). This study aims to ascertain the prevalence of orthoptic defects and refractive errors in a cohort of children affected by ASD, to provide an overview of the visual-motor status in our cohort of ASD children. This is an exploratory study. Therefore, the emerged statistical significances should be interpreted as hypothesis-generating rather than confirmatory, as explained in the literature (20).

## Methods

This retrospective study was conducted at the Pediatric Ophthalmic and Neuropsychiatric Units of the University Hospital of Verona, Italy. The charts of patients affected by ASD and admitted between 2018 and 2022 to the Pediatric University Hospital of Verona (Ospedale della Donna e del Bambino) were collected and reviewed. The inclusion criteria were the following: (1) Diagnosis of autism according to the Diagnostic and Statistical Manual of Mental Disorder 5th edition (DSM-5) (1, 21). (2) Availability of complete ophthalmologic examination, including monocular best-corrected visual acuity (BCVA) assessment, cycloplegic refraction, slit-lamp anterior segment examination, fundus ophthalmoscopy, stereopsis evaluation, complete ocular motility, and strabismus examination. (3) Absence of syndromes, brain lesions, or major sensory impairments.

Healthy controls of the same age range were selected from children who underwent complete ophthalmologic screening in our Clinic.

BCVA was assessed using age-appropriate tests, Lea symbol charts were used for preschool children and the Snellen Acuity charts for literate ones.

To avoid the effect of age on the visual acuity readings, and thus make comparable patients of different ages, we created 2 categories of visual acuity: group 1 (within the range for age) included eyes with a VA comprised in the 95% (±2SD) prediction interval for age; group 2 (under the range for age) included eyes with a VA inferior to prediction interval for age (<−2 SD) (22).

Ocular motility (fixation, pursuit, saccades and convergency), strabismus, and stereopsis were evaluated by expert pediatric orthoptists at our Clinic. Strabismus was assessed by the Hirschberg test and cover-uncover test. Lang test II was used for stereopsis assessment, the pointing of elements was considered sufficient, in relation to the cognitive status of some patient. The adequate stereopsis was defined as the localization of the three figures. The absence of stereopsis was defined as the inability to localize none of the three figures. Stereopsis deficit is the ability to localize at least one or two pictures (immature stereopsis). Normal convergence is defined as the ability to converge until a small, structured stimulus reaches the root of the nose, less than 5 cm. Convergence deficit is the ability to converge between 5 and 10 cm from the root of the nose. Beyond 10 cm it is defined as absence of convergence.

The complete ocular examination was conducted by trained pediatric ophthalmologists following the “Pediatric Eye Evaluation Preferred Practice Patterns” of the American Academy of Ophthalmology (AAO) (23). Firstly, pupil reaction was evaluated. Anterior segment was examined with the slit lamp, while fundus with the indirect ophthalmoscope, after pupil dilatation.

All patients underwent cycloplegic refraction. Refractive errors were detected and corrected. Refractive errors, normalized for age, were defined in accordance with AAO guidelines (23) (Table 1).

**Table 1:** Orthoptic evaluation in ASDs patients and controls.

	ASD children (*n* = 203)	Controls (*n* = 50)
Deficit of eye motility, *n* (%)	3 (1.5%)	0 (0.0%)
Convergence insufficiency, *n* (%)	26 (12.8%)	4 (8.0%)
Strabismus, *n* (%)	9 (4.4%)	3 (6.0%)
Stereopsis deficit, *n* (%)	64 (31.5%)	1 (2.0%)
Other ocular anomalies	1 (0.5%)	1 (2.0%)
Total of affected patients	103 (47.8%)	9 (18.0%)

Difference between cases and controls ascertained comparing overall proportion of subjects with at least one pathology (2×2 chi squared test: adjusted *p* < 0.001).

Categorical variables were reported by the frequency with percentage. Quantitative data were reported by mean with SD, not normally distributed quantitative data by median with interquartile range (IQR). Orthoptic and refractive defects were analyzed by comparing the proportions of subjects with at least one defect and using 2×2 chi-squared tests. LogMAR level (normal, subnormal, pathologic) were compared by Mann–Whitney U-test. Because logMAR was measured in each eye, the comparison was performed by considering the worst logMAR value within each subject. To address alpha inflation and preserve the robustness of the study’s conclusions, the statistical significances of the three tests were adjusted using Bonferroni correction by tripling the value of the *p*-values.

Statistical analysis was carried out with Stata software version 14 (StataCorp, College Station, TX). Statistical significance was set at a *p*-value < 0.05.

## Results

A total of 253 patients (203 ASD and 50 healthy controls) were included in the study.

Among the 203 ASD patients (406 eyes), 157 were males (77.3%) and 46 were females (22.7%). The mean age was 6.2 years (range 3–17). 50 healthy subjects (100 eyes), 32 males (64.0%) and 18 (36.0%) females, mean age 6.4 (range 4–15), were recruited in the control group.

According to the DSM-5, 36% of ASD patients (73 patients) were high functioning (mild, grade 1), 41.5% (84 patients) were medium functioning (moderate, grade 2), and 22.5% (46 patients) were low functioning (severe, grade 3).

Results about orthoptic evaluation, including ocular motility, convergence, strabismus, and stereopsis are collected in Table 1.

As shown in Table 1, a higher overall number of total orthoptic defects were observed in the ASD group, in comparison with the control group. Specifically, defects are present in 47.8% of ASD subjects and in 18% of controls (2×2 chi squared test: adjusted *p* < 0.001). This difference is mainly attributable to the number of stereopsis deficit present in cases (64 subjects, 31.5%) compared to controls (one subject, 2.0%). On the contrary, 3 out of 50 controls (6.0%) showed strabismus while only 9 cases (4.4%) did.

Analysis of refractive errors is shown in Table 2.

**Table 2:** Comparison of refractive defects in the right (RE) and left (LE) eyes between ASDs patients and controls.

	RE in ASD children (*n* = 203)	RE in controls (*n* = 50)	LE in ASD children (*n* = 199)	LE in controls (*n* = 50)
Emmetropia, *n* (%)	114 (56.2%)	37 (74.0%)	113 (56.8%)	37 (74.0%)
Hyperopia, *n* (%)	59 (29.1%)	6 (12.0%)	55 (27.6%)	5 (10.0%)
Myopia, *n* (%)	7 (3.6%)	3 (6.0%)	7 (3.5%)	3 (6.0%)
Astigmatism, *n* (%)	23 (11.3%)	4 (8.0%)	24 (12.1%)	5 (10.0%)
Eyes with at least one refractive error, *n* (%)	89 (43.8%)	13 (26.0%)	86 (43.2%)	13 (26.0%)
Subjects with at least one refractive error, n (%)	ASD group		Controls	
	91 (44.8%)		14 (28.0%)	

Difference between cases and controls ascertained comparing overall proportion of subjects with at least one defect (2×2 chi squared test: adjusted *p* = 0.09).

As shown in Table 2, 44.8% of ASD subjects shows at least one refractive defect, compared to 28% of controls. This difference does not reach statistical significance (2×2 chi squared test: adjusted *p* = 0.09). Hyperopia is the most represented refractive error in ASD subjects with more than a quarter of them being affected.

Table 3 shows the BCVA values in right and left eyes, in ASD patients and controls. In 97 of 203 ASD patients (47.8%) at least one eye had BCVA below the range for age. Otherwise, only one (2%) subject within the control group was below range. A statistically significant difference (2×2 Fisher’s exact test: adjusted *p* < 0.001) was detected.

**Table 3:** Best corrected visual acuity (BCVA) in right (RE) and left (LE) eyes in ASD and control group.

	BCVA RE		BCVA LE	
	ASD (*n* = 203)	Controls (*n* = 50)	ASD (*n* = 203)	Controls (*n* = 50)
In range, *n* (%)	122 (60.1%)	50 (100%)	130 (64.0%)	49 (98.0%)
Below range, *n* (%)	81 (39.9%)	0 (0.0%)	73 (36.0%)	1 (2.0%)
Subjects with at least one eye below range, *n* (%)	ASD group		Controls	
	97/203 (47.8%)		1/50 (2.0%)	

Difference between cases and controls ascertained by comparing in-range/below range age-corrected logMAR value (Fisher’s exact test–test: adjusted *p* < 0.001).

## Discussion

There has been a recent rise of interest in the nonsocial symptoms present in ASD, particularly, since there is a correlation between ASD and motor disability and the known sensory processing difficulty, the visual-motor aspects of ASD were investigated and it seems that there is a link between ASD and visual-perceptive/visual-motor deficit (24, 25).

This study aimed to rate the prevalence of orthoptic and ophthalmic defects in a cohort of children diagnosed with ASD, to understand the visual-motor status of these patients. For this purpose, data related to the visual-motor process in these patients were analyzed, as collected during standard eye examinations at the Pediatric Ophthalmic Unit of the University of Verona.

The statistical significance achieved in the analysis of the orthoptic evaluations data suggested a higher prevalence of convergence and stereopsis deficits in ASD patients, compared with the control group. This result supports what was previously analyzed in the introduction. It has been noted in previous studies that children with neurodevelopmental disorders have poor coordination (26). The alteration of stereopsis in part could provide an explanation for the motor and coordination embarrassment found in these patients (27). Indeed, it has been suggested that at the origin of some characteristic features of ASD there may be a “dorsal stream vulnerability,” i.e., an alteration of the dorsal stream network (24). The vulnerability of the dorsal neural stream can also impair attention, executive function, and spatial navigation. It seems that these traits of ASD are related to an inability to update perception from previous experience (28). It is unclear whether this general deficit extends to depth and stereo-disparity processing. An alternative theory proposes that autistic individuals have enhanced perceptual function (EPF) in early associative areas of sensory processing like visual discrimination, resulting in more locally oriented processing (29). For this reason, higher-order processing is not always employed or mandatory in ASD, when a task can be performed using lower-level perceptual processing. It is for this reason that, when presented with complex, fast-moving social stimuli, strong attention to low-level perceptual features can result in information overload and an inability to pay attention to the relevant visual cues (28, 30).

The detected convergence deficit may also be the result of impaired extrinsic eye movement coordination (31). Although the evidence is limited, it is thought that coordination difficulties may underpin the behavioral changes typical of ASD (32, 33). In fact, motor skills are closely interconnected with social ones. Both fine (e.g., gaze, handling objects) and gross (e.g., gesturing, walking) movements are important for maintaining attention and for imitation, both of which are important for social interaction (28, 34, 35). Depth perception and stereopsis play a key role in the management of the peri personal space and in the interaction with the other. Stereopsis can underlie deficits in motor abilities and can impact social skills (30, 36).

In this study, cycloplegic refraction revealed a difference between ASD children and controls, although not significant. Hyperopia was the most represented refractive error. Astigmatism and myopia were also reported, albeit with lower incidence. These findings agree with previous results (37–41). We corrected both the visual acuity and the refractive error for the patient’s age. We therefore considered only whether visual acuity was normal or below normal range for age. Concerning visual acuity, we found no significant difference between ASD and controls in right and left eyes. Approximately 90% of the patients showed normal visual acuity, although 30% of these were at the lower limits of normal. The explanation for this result is that 64% of the patients were with ASD grade 2 or 3, who have some degree of intellectual disability, which may have an impact in performing the test. Therefore, our study supports the hypothesis that visual acuity is not compromised in children with ASD (42). This finding confirms what was previously stated by Milne et al., that many aspects of vision, including visual acuity, are unaffected in ASD patients (43). BCVA evaluation may represent a challenge during pediatric examinations, requiring high levels of interaction between the child and the examiner. Difficulties especially could manifest during ASD patients’ evaluation, when collaboration between doctor and patient easily interrupt (37). In this scenario, the establishment of a comfortable outpatient environment and a trustful relationship with the patient require a high level of clinical experience. For this reason, it is fundamental to refer ASD children to a specialized Pediatric Centre, where collaboration between ophthalmologists and neuropsychiatrists is tight.

A strength of this research is the tight selection of cases, the sample size despite being a single-center study and the execution of tests by highly specialized professionals in pediatric ophthalmology and orthoptics. Indeed, many markers are evaluated, giving a complete scenery of the visual-motor involvement in our sample.

However, the evidence is not adequate to definitively describe the prevalence rate of visual-motor conditions in children with ASD (18) and this study cannot demonstrate the relationship between stereopsis and convergence deficits and impaired motor and social skills in patients with ASD. For this reason, our study represents a contribution to this field and further studies are needed to investigate these aspects.

The weakness point of our study is the lack of integration of our evaluations with objective tests, as electrophysiological ones or electronic saccade tracking (3). This aspect may need further investigation.

## Conclusion

In conclusion, the early assessment of sensory–motor abilities must be considered as crucial in follow-up programs for children at risk of ASD. Convergence insufficiency, stereopsis deficit and hyperopia are the most observed ocular conditions, which need particular attention. These findings are consistent with the known alterations of the dorsal stream network and the impairment of sensory processing in ASD. As a result, a complete ophthalmic evaluation is highly recommended in children with ASD, to guarantee early detection and treatment of possible visual-motor defects. Therefore, this study supports the evidence of limited visual impairment in children with ASD in our cohort, although it cannot be ruled out that some degree of visual developmental delay may be present. Further studies in this field are needed.

## Data availability statement

The raw data supporting the conclusions of this article will be made available by the authors, without undue reservation.

## Ethics statement

The studies involving human participants were reviewed and approved by Comitato Etico delle Province di Verona e Rovigo. Written informed consent to participate in this study was provided by the participants’ legal guardian/next of kin.

## Author contributions

All authors contributed to the study conception and design. Material preparation and data collection were performed by RL, LG, and FA. Analysis were performed by GG and LG. The first draft of the manuscript was written by RL and FA and all authors commented on previous versions of the manuscript. All authors contributed to the article and approved the submitted version.
